# Modeling of Non-Small Cell Lung Cancer Volume Changes during CT-Based Image Guided Radiotherapy: Patterns Observed and Clinical Implications

**DOI:** 10.1155/2013/637181

**Published:** 2013-10-24

**Authors:** Hiram A. Gay, Quendella Q. Taylor, Fumika Kiriyama, Geoffrey T. Dieck, Todd Jenkins, Paul Walker, Ron R. Allison, Paolo Ubezio

**Affiliations:** ^1^Department of Radiation Oncology, Washington University School of Medicine, 4921 Parkview Place, Campus Box 8224, St. Louis, MO, 63110, USA; ^2^Maine Medical Center, Southern Maine Radiation Therapy Institute, 22 Bramhall Street, Portland, ME 04102, USA; ^3^Department of Radiation Oncology, Pocono Medical Center, 206 East Brown Street, East Stroudsburg, PA 18301, USA; ^4^Nash Cancer Treatment Center, 2450 Curtis Ellis Drive, Rocky Mount, NC 27804, USA; ^5^Department of Medical Oncology, The Brody School of Medicine, East Carolina University, 600 Moye Boulevard, Greenville, NC 27834, USA; ^6^21st Century Oncology, 801 W.H. Smith Boulevard, Greenville, NC 27834, USA; ^7^Laboratory of Anticancer Pharmacology, Department of Oncology, IRCCS - Istituto di Ricerche Farmacologiche Mario Negri, Via La Masa 19, 20156 Milan, Italy

## Abstract

*Background*. To characterize the lung tumor volume response during conventional and hypofractionated radiotherapy (RT) based on diagnostic quality CT images prior to each treatment fraction. *Methods*. Out of 26 consecutive patients who had received CT-on-rails IGRT to the lung from 2004 to 2008, 18 were selected because they had lung lesions that could be easily distinguished. The time course of the tumor volume for each patient was individually analyzed using a computer program. *Results*. The model fits of group L (conventional fractionation) patients were very close to experimental data, with a median Δ% (average percent difference between data and fit) of 5.1% (range 3.5–10.2%). The fits obtained in group S (hypofractionation) patients were generally good, with a median Δ% of 7.2% (range 3.7–23.9%) for the best fitting model. Four types of tumor responses were observed—Type A: “high” kill and “slow” dying rate; Type B: “high” kill and “fast” dying rate; Type C: “low” kill and “slow” dying rate; and Type D: “low” kill and “fast” dying rate. *Conclusions*. The models used in this study performed well in fitting the available dataset. The models provided useful insights into the possible underlying mechanisms responsible for the RT tumor volume response.

## 1. Introduction

Understanding lung tumor volume changes during radiotherapy may one day help radiation oncologists optimize dose fractions and radiosensitizing strategies for individual patients. A small number of studies have described lung tumor volume changes during conventionally fractionated external beam radiotherapy [1–5]. Lung tumor changes during hypofractionated radiotherapy, especially when delivered in short courses (≤2 weeks), are less well understood. Four studies have included patients receiving hypofractionated treatments to varying degrees [6–9], and one study included only large fraction (>7.5 Gy) hypofractionated RT [8].

Normal cell proliferation is based on the cell cycle, through which cells duplicate their genome and protein mass, enter mitosis, and divide. Cancer is essentially a proliferative disorder resulting from uncontrolled cell cycling of transformed cells which encode modified regulatory proteins. For a given normal tissue or cancer, a fraction of the cells is physiologically out of the cycle (quiescent, or “*G*0” cells), although they may enter the cycle with suitable signals. The expansion of a cell population depends on the balance between three phenomena: cell cycling, the quiescence/cycling balance, and cell death. This balance is strictly controlled in normal tissues but is dysregulated in cancer due to reduced cell death or quiescence and/or increased cell cycling which causes accelerated expansion of the cell population. Notably, proliferation features are variable within a cell population given that the cell cycle length (*Tc*) is highly heterogeneous. Mathematical modeling of this phenomenon has a long history [10], and it is theoretically grounded by several works [11–14]. The tumor growth model adopted here is not needlessly complicated while accounting for the basic processes of cell cycling, quiescence, and cell loss [15]. The model was modified to account for treatment efficacy, based on a simple description of cell killing and resistance, and was proven suitable for fitting time courses of tumor volumes when measured by calipers during neoadjuvant breast cancer chemotherapy [16]. In this study, we use this mathematical model to interpret time courses of nonsmall cell lung cancer (NSCLC) tumor volumes measured by CT scans during conventionally fractionated or hypofractionated radiotherapy (RT).

## 2. Materials and Methods

A retrospective review of our IGRT database at East Carolina University identified 26 consecutive patients who had received CT-on-rails IGRT (CTVision, Siemens, Malvern, PA) to the lung from September of 2004 to November of 2008. The CT-on-rails system consists of a CT scanner and a linear accelerator opposing each other in the treatment vault and sharing the patient couch. The daily CT was of diagnostic quality and was always obtained prior to treatment.

Of the 26 patients identified, 18 were selected because they had lung lesions that could be easily distinguished from other mediastinal structures and also underwent daily IGRT during treatment. The lesions were predominantly spherical in nature and surrounded by lung parenchyma which eased the contouring process. Twelve patients were treated with fractions ≤600 cGy, and 5 (Table 1, patients L1–L5, group L = long) met the criteria for this analysis. Fourteen patients were treated with fractions ≥1000 cGy, and 13 (Table 1, patients S1–S13, group S = short) met the criteria for this analysis. Group S, the hypofractionation group, completed treatment in less than 14 days.

Patient S9, with an initial tumor volume of 0.4 cc, had close to a fivefold increase in volume in the three days that elapsed from the penultimate fraction to the last fraction due to atelectasis and was excluded from the analysis.

The planning CT was obtained with a 3 mm slice thickness and with the patient in the supine position. To minimize human bias, the tumor lesions were contoured in the planning CT using the CMS Xio (Release 4.34.02, St. Louis, MO) treatment planning software's (TPS) autothreshold contouring tool (*W* = 500, *L* = 0). The TPS reports the tumor volume based on all the contours comprising a given tumor. The same procedure was done to estimate the tumor volume on the daily treatment CT, and additional diagnostic CT scans, pre- or posttreatment, when available.

### 2.1. Model Description

**Figure 1:**
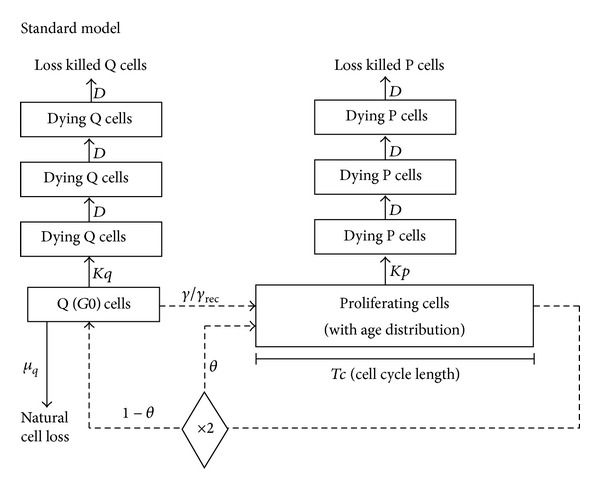
Block diagram of the proliferation model. A fraction *θ* of newborn cells directly enters the cycling stage, while the others (1 − *θ*) become quiescent (Q or *G*0). Quiescent cells either die (with a rate *μ*_0_) or reenter into the cycling stage (with a “recycling” rate *γ*). The parameter *γ* is zero or very low (say no more than 0.01, meaning 1% of quiescent cells become cycling per day—otherwise these cells were not “quiescent”). Cycling cells traverse the cell cycle (G_1_ + S + G_2_ M) in an average time *Tc*, after which they divide generating two newborn cells. The parameters *θ* and *μ*_*q*_ are dependent on the input values of *Td*, *GF*, *Tc*, and *γ*. Modeling of the proliferation is based on the general mathematical theory of proliferating cell populations (see Ubezio and Cameron [16] and the references therein).

The time course of the tumor volume for each patient was individually analyzed using a previously described [16] computer program that allows for testing different models of tumor growth and therapy efficacy (Figure 1). The tumor growth model is based on the equilibrium between cycling cells, quiescent cells, and cell loss, resulting in exponential growth. Although growth retardation is expected when the tumor volume increases, the exponential growth model is a very reasonable approximation during an observation range of a few weeks to months. The tumor growth model is based on the age-structured cell cycle mathematical theory of Bertuzzi et al. [15]. Tumor growth is defined by four parameters: doubling time (*Td*), growth fraction (*GF*, i.e., the fraction of cycling cells), cell cycle duration (*Tc*), and the rate “*γ*” at which quiescent cells reenter the cycle. Other model parameters like the cell loss rate (*μ*_*q*_), potential doubling time (*T*_pot_), and the fraction of newborn cells bypassing *G*0  (*θ*) are calculated from the previous four parameters [16]. The equations for the number of cycling (*Np*) and quiescent (*Nq*) cells in our discrete model of exponential growth were the following:

(1) Np(t)=Np(t−Δt)+θ×2u×Np(t−Δt)Δt′−u×Np(t−Δt)Δt′+γ×Nq(t−Δt)Δt′,Nq(t)=Nq(t−Δt)+(1−θ)×2u×Np(t−Δt)Δt′−(γ+μq)×Nq(t−Δt)Δt′,

where *u* is the rate at which cycling cells end the cell cycle, linked to *Tc* and *Td* via the following relationship: *u* = ln⁡(2)/(*Td* × (*e* ^ln⁡⁡(2)×(*Tc*/*Td*)^ − 1)), and Δ*t*′ = (*e* ^ln⁡⁡(2)×(Δ*t*/*Td*)^ − 1)/(ln⁡(2)/*Td*) which connects the discrete model, acting with step-time Δ*t*, to the continuous model (Δ*t*′ → Δ*t*, when Δ*t* ≪ *Td*). In this study we set Δ*t* = 1 day.

The dependent parameters are provided by the following formulae, derived from the described theory [15]:

(2) $$\begin{array}{c} T_{\text{pot}}=\frac{\left(e^{\ln\left(2\right)\times (Tc/Td)}-1\right)}{GF}\times Td,\\ \mu_{q}=\frac{\ln\left(2\right)\times \left(1/T_{\text{pot}}-\left(1/Td\right)\right)}{\left(1-GF\right)},\\ \vartheta =\frac{e^{\ln\left(2\right)\times (Tc/Td)}}{2}+\gamma \times \frac{\left(e^{\ln\left(2\right)\times (Tc/Td)}/2\right)-1}{\left(\ln\left(2\right)/Td\right)+\mu_{q}}. \end{array}$$

Once *N*(0) is given with *Td*, *Tc*, *GF*, and *γ*, the time-zero state variables are simply *Np*(0) = *GF* × *N*(0) and *Nq*(0) = (1 − *GF*) × *N*(0). The ratio *Np*/(*Np* + *Nq*) remains equal to *GF* over time. The above equations describe the dynamics of the unique asynchronous exponential growth associated with a set of independent parameters. The choice of using parameters which are observable is convenient because it allows to use their direct measure, when available, or to verify the biological validity of the models. Data from the literature suggests that typical values for *Td*, *GF*, and *T*_pot_ are 100 days [17–20], 0.2 [21, 22], and 8 days [18, 23], respectively, with a wide interpatient variability of kinetic parameters in NSCLC. In the present study, these four parameters were individually estimated within the range described in the literature.

The equations are based on numbers of tumor cells, while the data was tumor volume. We assumed that the measured tumor volume was directly proportional to the number of tumor cells, including those dying, assuming 10^9^ cells = 1 cm^3^. However, the specific value of the proportionality constant does not affect the results. The following four models of radiation efficacy were considered.

*Minimal (M) Model*. The M, or simplest, model has no differential efficacy between cycling and quiescent cells. The parameters of the model are *K* and *D*. The parameters of quiescent cells (*θ*, *γ*, and *GF*) become irrelevant. The effect is described by the percentage of cells killed (*K*) by a single fraction of radiotherapy. At the times of treatment (*t*_tr⁡_), the situation immediately before (*t*_tr⁡_−) the treatment is considered separately from that immediately after (*t*_tr⁡_+) the treatment, and the number of surviving, cycling, and quiescent cells is reduced according to the following:

(3) $$\begin{array}{c} Np\left(t_{\mathrm{tr}}+\right)=\left(1-\frac{K}{100}\right)\times Np\left(t_{\mathrm{tr}}-\right),\\ Nq\left(t_{\mathrm{tr}}\right)=\left(1-\frac{K}{100}\right)\times Nq\left(t_{\mathrm{tr}}-\right). \end{array}$$

“Killed” cells are not immediately removed from the tumor, but they are lost when they complete a three-stage dying process with a single stage rate “*D*.” In this way, the time to definitive loss is a random variable whose probability density function is the convolution of exponentials, also known as the Erlang function. For example, *D* = 0.75 means 75% of cells in dying stage I move to dying stage II in one day, 75% in stage II move to stage III, and 75% in stage III are definitely lost. With *D* = 0.75 one can calculate that the dying process takes an average four days before the disappearance of the cells, six days when *D* = 0.5, fifteen days when *D* = 0.2, and immediate (i.e., <3 days) loss when *D* = 1.

The following equations hold for the number of cells in these stages (*N*_*d*1_, *N*_*d*2_, and *N*_*d*3_, resp.):

(4) $$\begin{array}{c} N_{d1}\left(t\right)=\left(1-D\right)\times N_{d1}\left(t-\Delta t\right),\\ N_{d2}\left(t\right)=\left(1-D\right)\times N_{d2}\left(t-\Delta t\right)+D\times N_{d1}\left(t-\Delta t\right),\\ N_{d3}\left(t\right)=\left(1-kD\right)\times N_{d3}\left(t-\Delta t\right)+D\times N_{d2}\left(t-\Delta t\right), \end{array}$$

while at treatment times killed cells add to cells in the first stage:

(5) $$\begin{array}{c} N_{d1}\left(t_{\mathrm{tr}}+\right)=N_{d1}\left(t_{\mathrm{tr}}-\right)+\frac{K}{100}\times \left(Np\left(t_{\mathrm{tr}}-\right)+Nq\left(t_{\mathrm{tr}}-\right)\right). \end{array}$$

*Standard (St) Model*. The St model includes the differential efficacy between cycling and quiescent cells. The parameters of the model are *Kp*, *Kq*, and *D*. The effect is described by the percentage of cycling (*Kp*) and quiescent (*Kq*) cells killed by a single fraction. Thus, at treatment times the number of cycling and quiescent cells is given by the following:

(6) $$\begin{array}{c} Np\left(t_{\mathrm{tr}}+\right)=\left(1-\frac{Kp}{100}\right)\times Np\left(t_{\mathrm{tr}}-\right),\\ Nq\left(t_{\mathrm{tr}}\right)=\left(1-\frac{Kq}{100}\right)\times Nq\left(t_{\mathrm{tr}}-\right). \end{array}$$

*Recruitment (REC) Model*. The REC model allows a burst of quiescent cells to enter the cell cycle at a specified interval during treatment with a “recruitment rate” which overperforms the pretreatment rate *γ*. This model reflects the concept of accelerated repopulation of the cycling tumor cell pool. In normal conditions, the rate *γ* at which quiescent cells go into the cycling stage is zero or very low. However, it is possible that a fraction of quiescent cells is stimulated to proliferate as a consequence of an RT treatment. This phenomenon is modeled here assuming that for a short period after treatment, the value of *γ* becomes higher than 0.01, and the parameter is renamed “*γ*_rec_.” The parameters of the model are *Kp*, *Kq*, *D*, and *γ*_rec_.

*Resistance (RES) Model*. The RES model includes the presence of radioresistant cells. The computer program allows for testing two different resistance models: “initial resistance,” assuming a subpopulation of resistant cells is already present at the start of treatment (Rini being the fraction of resistant cells at that time), and “induced resistance” assuming a fraction of cells (Rind) becomes resistant after each RT treatment. The same growth equations hold for sensitive and resistant cells. The number of cycling and quiescent resistant cells is indicated as *Nrp*(*t*) and *Nrq*(*t*), respectively.

In the case of resistant cells at the time of treatment, the starting values are given by the following:

(7) $$\begin{array}{c} Np\left(0\right)=N\left(0\right)\times GF\times \left(1-\text{Rini}\right),\\ Nq\left(0\right)=N\left(0\right)\times \left(1-GF\right)\times \left(1-\text{Rini}\right),\\ Nrp\left(0\right)=N\left(0\right)\times GF\times \text{Rini},\\ Nrq\left(0\right)=N\left(0\right)\times \left(1-GF\right)\times \text{Rini}. \end{array}$$

In the case of induced resistance, the equations at the times of treatment are modified as follows:

(8) $$\begin{array}{c} Np\left(t_{\mathrm{tr}}+\right)=\left(1-\frac{Kp}{100}\right)\times Np\left(t_{\mathrm{tr}}-\right)\times \left(1-\text{Rind}\right),\\ Nq\left(t_{\mathrm{tr}}+\right)=\left(1-\frac{Kq}{100}\right)\times Nq\left(t_{\mathrm{tr}}-\right)\times \left(1-\text{Rind}\right),\\ Nrp\left(t_{\mathrm{tr}}+\right)=Nrp\left(t_{\mathrm{tr}}-\right)+\left(1-\frac{Kp}{100}\right)\times Np\left(t_{\mathrm{tr}}-\right)\times \text{Rind},\\ Nrq\left(t_{\mathrm{tr}}+\right)=Nrq\left(t_{\mathrm{tr}}-\right)+\left(1-\frac{Kq}{100}\right)\times Nq\left(t_{\mathrm{tr}}-\right)\times \text{Rind}. \end{array}$$

The overall number of tumor cells at a time “*t*” is the sum of sensitive cycling, sensitive quiescent, resistant cycling, resistant quiescent, and dying cells, namely, the following:

(9) N(t)=Np(t)+Nq(t)+Nrp(t)+Nrq(t)+Nd1(t)+Nd2(t)+Nd3(t).

Both the model M and the standard model can be modified to include radioresistance, obtaining the “M Res” and “St Res” models. Parameters for the models are *K* (or *Kp*, *Kq*), *D*, and Rini (or Rind).

The framework of our computer program allows building even more complex models; for example, combining recruitment and resistance models or models with non-constant *K*. These more complex models were not required to fit the data of the present study.

Using a principle of parsimony we first tested simpler models, introducing more complex effects only when they allowed improving the fitting in a statistically significant way (likelihood ratio test statistics). The program outputs the complete time course of the tumor volume. In some instances, alternative models were reported too, not significantly worse than the best one.

### 2.2. Fitting Procedure and Sensitivity Analysis

The standard model has eight independent parameters: *V*0 (the tumor volume at the beginning of treatment), *Td*, *Tc* (or *T*_pot_), *GF*, *γ*, *Kp*, *Kq*, and *D*. The minimal model requires four parameters: *V*0, *Td*, *γ*, *K*, and *D*. An additional parameter is included in the M Rec and St Rec models (*γ*_rec_) and in the M Res or St Res models (Rini or Rind). Except in the case of pt L1, where we adopted the value *γ* = 0.01 in the model Strec, preliminary modeling led us to set *γ* = 0 because higher values did not improve the fits.

An estimate of the other growth parameters (*Td*, *Tc*, *GF*) was made possible by including into the fit the pretreatment period between the planning CT scan and the start of treatment. When the prtreatment volume was not available, we considered two models with *Td* equal to 25 or 150 days.

For each model, parameters were optimized with a constrained, nonlinear fitting procedure, maximizing the likelihood function (*L*) of the logs of tumor volumes, with a Gaussian distribution of data errors taking their standard deviation as a parameter as described before [16]. Likelihood-based 95% confidence intervals for each parameter were obtained by raising or lowering its value until *L* was reduced to the value of log⁡(*L*) = log⁡(*L*_best_) − *χ*_0.05,1_^2^/2 [24].

### 2.3. Implementation

All analyses were done with a computer program using Excel (Microsoft, Redmond, WA) with its standard features (Visual Basic and Solver). A user-friendly interface graphically displays the tumor volume simulation. The program is available for noncommercial purposes.

## 3. Results

The individual patient, tumor, and treatment characteristics are detailed in Table 1. The median tumor volume was 3.6 cc (range 0.4–161.1). The time courses of tumor volume measurements of the 17 patients who received fractionated (group L, 5 patients) or hypofractionated (group S, 12 patients) RT were fitted with the M and St models. The simpler M model was adopted if the fitting was similar between both. Similarly, the even more complex RES or REC models were adopted only when they produced a significant improvement over the fits obtained with the M or St models. Model parameters are summarized in Table 2. The details of the best fit models for the tumor volume time course for each patient are reported in Table 3.

The model fits of group L patients were very close to experimental data, with a median Δ% (average percent difference between data and fit) of 5.1% (range 3.5–10.2%) for the best fitting model. The fits obtained in group S patients were generally good, with a median Δ% of 7.2% (range 3.7–23.9%) for the best fitting model and with 9 out of 12 cases below 10%. Abrupt increases or decreases of volume measures were sometimes observed in group S but not in group L.

Patients with similar response to treatment were grouped according to tumor cell killing as “high” (*K* or *Kp* ≥ 5% in group L, *K* or *Kp* ≥ 35% in group S) or “low” (not “high”), and the speed of the dying process as “fast” (*D* ≥ 0.5) or “slow” (not “fast”). The responses to treatment were in turn typed as follows.

- Type A: “high” kill and “slow” dying rate.
- Type B: “high” kill and “fast” dying rate.
- Type C: “low” kill and “slow” (or not detectable) dying rate. Poor responders.
- Type D: “low” kill and “fast” dying rate. Poor responders.

**Figure 2:**
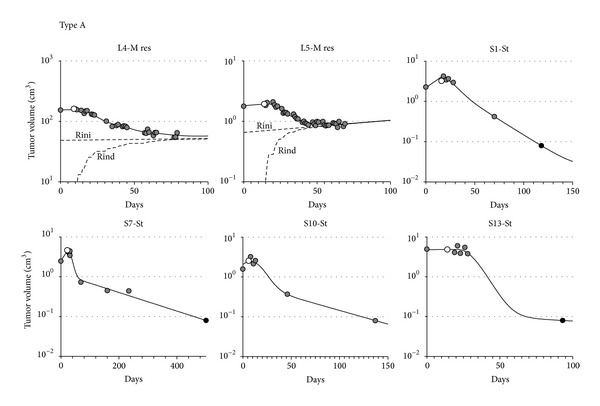
Cell number versus days for patients with a Type A response (high percentage of killed cells and slow dying rate). Gray circles: data points; white circle: treatment starts; black circles: data assumed equal to the CT detection limit (when they are actually below it); continuous line: best fit model; dashed lines: subpopulation of resistant cells, either with the initial (Rini) or the induced (Rind) resistance models.

**Figure 3:**
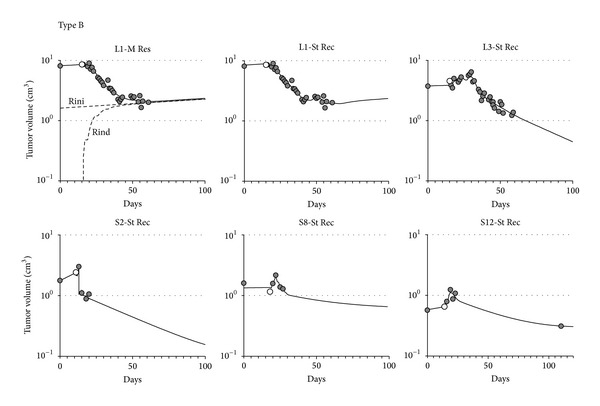
Cell number versus days for patients with a Type B response (high percentage of cells killed and fast dying rate). Symbols are as in Figure 2.

**Figure 4:**
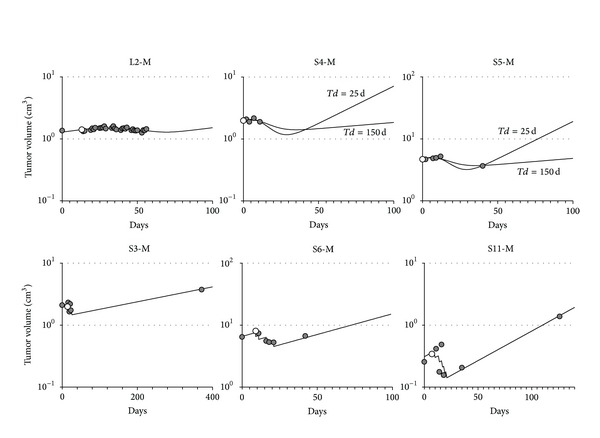
Cell number versus days for patients with Type C (low percentage of cells killed and slow dying rate) and Type D (low percentage of cells killed and fast dying rate) responses. Symbols are as in Figure 2.

Group L had two Type A responses, two Type B responses, and one Type C response. Group S had four Type A, three Type B, two Type C, and three Type D responses. Figures 2 to 4 report the data (circles) and the best fit model (continuous line) of all measured time courses.

### 3.1. Type A Response (Figure 2)

Type A response is characterized by a relatively high fraction of killed cells per fraction but a slow dying process, so that shrinkage of the tumor is delayed in time. Type A response was observed in patients L4, L5, S1, S7, S10, and S13. The model predicts a slow continuous shrinkage of the tumor that can continue after the end of treatment due to delayed loss of killed cells and natural cell loss when treatment has depleted the proliferating pool. Notice that in three patients (S1, S7, and S13) the last measurement was below the CT detection limit (indicated with a black circle). In these cases we conservatively assumed a measure equal to the CT detection limit, leading to possibly underestimating an effect that was already estimated as very high, which did not affect the classification. In the case of the L patients, the model estimates that each 2.5 Gy fraction killed 10% (patient L4) to 16% (patient L5) of sensitive tumor cells. However, the reduction of tumor mass was eventually exhausted, an effect that was modeled including a subset of resistant cells. The subpopulation of resistant cells (dashed lines in Figure 2) became prevalent within six to seven weeks. The two alternative models of resistance, that is, considering resistant cells present from the beginning (Rini) or induced by treatment (Rind), performed similarly in fitting the data so that the issue of the origin of resistance remained unanswered with the available data. The “initial resistance” estimates that about one third of the cells were initially insensitive to treatment in both cases, while the “induced resistance” model estimates that 3% (patient L4) to 6% (patient L5) of sensitive cells became resistant after each treatment fraction.

A higher shrinkage was reached in the S patients, without evidence of resistance. In these cases the standard model fitted the data significantly better than the minimal model and suggested that each 10 Gy exposure was able to kill ≥90% of cycling cells and a variable amount of quiescent cells ranging from 5% (patient S1) to 53% (patient S13).

### 3.2. Type B Response (Figure 3)

Type B is characterized by a faster tumor response and high killing rates. Volume time courses in patients L1, L3, S2, S8, and S12 are representative of this kind of response. A common landmark of the Type B response was a recruitment of cells into proliferation, fitted by St Rec models, although an alternative model including resistance is also consistent with the data in patient L1. Recruitment is clearly shown in patient L3 in the first week of treatment when the tumor size increased at a much higher rate than it did in the pretreatment period. Only in the second week the volume started decreasing and produced a rapid shrinkage of the tumor mass.

In contrast, in the case of patient L1 the tumor started shrinking shortly after the beginning of treatment. Recruitment occurred late, during the fourth week of therapy. By analogy with patient L3, if the St Rec model is correct, one would expect that continuation of RT would have further reduced the tumor mass in patient L1. Although the recruitment model provides the best fit for patient L1, the resistance model was not significantly worse and estimated that the lack of further reduction of the tumor mass around the sixth week was due to the emergence of a subpopulation of resistant cells. In this case, prolongation of treatment may have been futile. As for the other models which include resistance, the data was insensitive to the kind of resistance applied, either initial, with about 20% resistant cells at the start of treatment, or induced, with 3% sensitive cells becoming resistant after each dose.

According to the group L models, each 2.5 Gy dose killed roughly 16% of tumor cells (patient L1, model M res, *K* parameter) or 30% (patient L1, model St Rec, *Kp* parameter) to 50% (patient L3, model St Rec, *Kp* parameter) of cycling tumor cells, being almost ineffective against quiescent cells.

Recruitment was also detected in group S, although the short followup of the available data in patients S2 and S8 prevented a robust confirmation of the prediction of tumor reduction after the end of treatment. Approximately 65% to 90% of cycling cells were killed by each 10 Gy dose, while quiescent cells were unaffected. The mechanism postulated by the model is that these tumors had a high fraction (>90%) of quiescent cells, initially, that were recruited into proliferation by the first or second 10 Gy dose. Subsequent 10 Gy doses were extremely effective in killing newly cycling cells and caused a significant reduction of the tumor mass. At the end of the treatment, the pool of cycling cells was so depleted that surviving quiescent cells were unable to repopulate it in a short time, and the tumor mass continued to decrease by natural cell loss, until a new equilibrium was restored at a much lower tumor mass.

### 3.3. Types C and D Responses (Figure 4)

Types C and D responses were characterized by low killing, with a slow (Type C) or high (Type D) dying process resulting in a poor control of tumor growth.

The time courses for patients L2, S4, and S5 are representative of Type C response, modeled with the minimal model, with only 2% (group L) and 10% (group S) cells killed per fraction, respectively. Only a modest variation of tumor volume was observed over the treatment period denoting a poor response. However, an alternative explanation exists in the L2 case: the neoadjuvant chemotherapy likely killed most of the tumor, and the residual scar tissue masked the actual tumor volume. In the case of the time courses of patients S4 and S5, the absence of a pretreatment measurement made it impossible to estimate the doubling time. Fits assumed either a short (25 days) or a long (150 days) doubling time, but in both cases the therapy was predicted as poorly effective. With the shorter *Td*, the estimated fraction of killed cells per fraction was somewhat higher, but the tumor restarted to grow rapidly after the end of treatment. In the case of S5, however, the patient received bevacizumab after RT, and this was not considered in modeling. Thus, long-term predictions of the real outcome of this patient were impossible due to the unknown effect of the drug.

Type D is represented only within group S, and the minimal model provided the best fit. Killing per fraction was 8–13% in S3 and S6, and it was somewhat higher in S11 (22%), but it was low considering the high dose per fraction (10 Gy). Due to the fast cell dying process, a reduction of tumor mass was achieved and seen during treatment. Immediately after treatment the tumor restarted growing with its unperturbed rate. In S6 and S11, a short doubling time was estimated by the model, so that the benefits of the treatment were readily lost in a few weeks.

## 4. Discussion

To our knowledge, this is the first study applying mathematical modeling to characterize RT lung tumor volume response using diagnostic quality CT images prior to each treatment fraction, and it is the only study characterizing tumor response in patients receiving 10 Gy × 5 over a 2-week course.

Other studies have described the magnitude of tumor regression during RT without modeling. Erridge et al. analyzed electronic portal images in 25 patients treated with 54 to 81 Gy in 2–2.25 Gy per fraction [7]. In 40% of the patients, the projected area of the tumor regressed by more than 20% during treatment in at least one projection. Fox et al. studied 22 patients with NSCLC treated with 2 Gy daily fractions to a median dose of 62 Gy (range 50–74) with 15 (68%) treated with concurrent chemotherapy. A mean decrease in the initial GTV of 30% by a nominal dose of 30 Gy and 43% by a nominal dose of 50 Gy was observed [3]. In another study of 8 patients where the treatment details were not specified, tumor volume reduction during treatment ranged from 20% to 71% (end-inspiration) and from 15% to 70% (end-expiration) in 4DCT scans [25]. In all these studies, however, a closer analysis of the time course of the tumor volume was not attempted.

The performance of a locally weighted regression method to predict tumor volume at a specific time in NSCLC was successfully explored by Seibert et al. [6]. Patients were treated at 2 to 2.5 Gy per fraction to 50 to 74 Gy, including those considered in a previous study by the same group [4]. In 18 lesions, the authors observed a mean decrease in volume of 2.2% per day (range 0.6 to 5.8 per day), while 2 lesions showed an increase of up to 2% per day. In this approach the patterns of the time courses of tumor volume were considered per se, focusing on a prediction algorithm, with parameters unrelated to the underlying biological phenomena.

In the present work we adopted a phenomenological modeling of tumor proliferation and efficacy of treatment. This modeling approach was previously proven feasible in rendering the time evolution of breast cancer during preoperative chemotherapy [16]. The proliferation process is simulated *in silico* depending on parameters which are associated with the main biological phenomena in play, that is, the interplay between cell cycling, quiescence, and loss for untreated tumors, to which the effects of treatment are superimposed. Our modeling enabled us to estimate the percentage of cells killed by single doses of radiation, which has been rarely attempted in the clinical environment, and may give important information on an individual patient's tumor responsiveness. A similar approach was used by Ribba et al. to model low-grade glioma treated with chemotherapy and radiotherapy [26]. These authors modeled tumor proliferation including quiescent cells and differential treatment efficacy within cycling and quiescent cell subpopulations, considering delayed death. These features are also shared with our model.

To account for quiescent cells, we adopted what we called the “standard model,” representing a compromise between the complexity of the phenomenon of tumor proliferation and the relative simplicity of the time courses of our volume data. Our modeling approach exploits the theory of an age-structured cell population to render the proliferation process on the basis of three, potentially measurable, parameters: doubling time, potential doubling time, and growth fraction, all taking advantage of the information available on these parameters in the lung cancer literature. A fourth parameter, giving the rate of reentering in cycle of quiescent cells, is not directly measurable, but best fitting of our data required a very low value for it, and it was eventually fixed to zero with a single exception.

For what concerns the treatment model, the information available* in vivo* is not sufficient to disclose all the details observable *in vitro*, like blocks in specific cell cycle phases or the balance between repair and apoptosis [27]. At the time of each dose administration, a fraction of cells destined to die was selected, only distinguishing the rate of killing in quiescent from cycling cells, and then we applied a delay process through which cells committed to die completed the death process. In order to model the, possibly slow, process of elimination of killed cells, we assumed that dying cells stop proliferating and pass through three stages, envisaging progressive degrees of damage, before they are definitively lost and cause a decrease in tumor volume [28].

The use of more complex models, in our opinion, was not justified, leading to overparametrization and the impossibility of estimating the model parameters' values. On the other hand, a simpler model would not allow us to properly evaluate the proliferation process, in keeping with the present-day biological knowledge of the cell cycle machinery driving it.

In addition, our modeling approach optionally includes other factors that may influence the tumor response, like a subpopulation of radioresistant cells or radiation-induced recruitment of quiescent cells into proliferation, which were considered only upon the failure of simpler models.

Ultimately, the selected model for each patient fits the measured time course of tumor volume with good precision, with differences between the measured volumes and the model predictions below 10% in most cases. Pretreatment and long term tumor volume measurements, which were available in most instances, were crucial for the accuracy of the model estimates. In particular, long-term measurements were of greater importance in group S, where the treatment course was relatively short, and tumor volume reductions were not always appreciated. In these cases, further weekly measurements after treatment could have helped characterize the tumor response more accurately.

The small size of the conventional and hypofractionated RT groups makes it impossible to compare them in a proper statistical sense, adjusting for relevant variables. For example, a higher number of short doubling time estimates were obtained within group S compared to group L, which could account for some of the differences between the groups. However, the estimate of parameters describing radiation efficacy (i.e., *K* and *D*) was robust and poorly sensitive to the uncertainty of the doubling time (not shown). Instead, *Td* is obviously an important variable in the resumption of tumor growth after treatment.

One limitation of this study was that the intra- and interindividual contouring reliability was not evaluated. However, since we used the autocontouring planning tool with a defined window level, variability should have been minimized. Moreover, predictions of the models rely not on single points but on the entire time course of measures, catching the overall trend of the variations of tumor volume and somewhat buffering the errors of the single measurements. The average discrepancy between the model and the data suggests that the typical error of a single measurement was in the 5–10% range. Despite the above potential limitations, interesting and potentially useful information could be retrieved by comparison of the best fit models of all patients. In most cases, single doses were efficient for killing tumor cells, with typically 90% of cycling cells killed by 10 Gy and 30–50% killed by 2 Gy, with only a few percent quiescent cells killed (only in three cases more than 5% quiescent cells were killed by a single fraction). This was sufficient to induce a sustained tumor regression continuing after the end of treatment unless a population of radioresistant cells emerged.

Data analysis could have been done in the framework of mixed-effect models, where individual profiles are generated from a population model according to a unimodal distribution of parameters' values, as successfully done by Ribba et al. [26]. We preferred instead a different perspective aimed at distinguishing different types of responses that are also modeled differently (e.g., with or without resistance), starting from the simplest model and increasing complexity only when the fitting significantly improves. Based on the types of responses that have been identified, further studies can be planned recruiting a number of patients per group suitable for population-based analyses. Our modeling approach enabled the recognition of four different types of response and the interpretation of the kinetics of cell proliferation underlying each of them. Response types were characterized not only by different percentages of killed cells per dose (model parameter *K*) but also by the velocity of the disappearance of killed cells (model parameter *D*). In the case of response Type A, characterized by high cell kill with slow dying rate, hypofractionated therapy seemed to be more effective, eventually producing a higher volume reduction than conventionally fractionated therapy. In patients receiving conventional RT, tumor volume was initially reduced, but after 3-4 weeks of therapy it reached a sort of plateau. This was modeled by introducing a subpopulation of radioresistant tumor cells, whose presence would not have been detectable until it prevailed over the subpopulation of sensitive cells, depleted by treatment. Several studies of mathematical modeling of resistance have been published, mainly focusing on the emergence of the phenomenon [29–31], with one based on time-course datasets from patients during/after the treatment of hematological cancers [32].

We compared two models of resistance: one model assuming that a subset of resistant cells was already present at the beginning of treatment and the other assuming that resistance was induced by treatment. Unfortunately the data were fitted well by both models, the former estimating that 20–40% of the cells were initially resistant and the other estimating that 3–6% of cells became resistant after each fraction. Thus data were consistent with the presence of resistance but were insensitive to the underlying mechanism. There are very few studies of induced radiation resistance in the literature, but at least one experiment suggested that this could exist in the context of crossing over from doxorubicin and radiation, and vice versa [33]. This may be an area worth investigating, and develop strategies to overcome resistance with targeted therapeutic agents.

In the cases we modeled including resistance, further long-term response in the posttreatment period cannot be excluded a priori, but the available data, up to the second month from treatment start, do not justify this hypothesis. Other studies suggesting that a very late response is possible mostly refer to combined radiochemotherapy and were not designed to recognize an intermediate plateau [1, 5].

No plateau was instead observed in Type A patients receiving hypofractionated RT, where the model predicts a continuous shrinkage of the tumor long after the end of the treatment. This effect was due not only to the delayed loss of cells killed during the treatment but also to the natural cell loss occurring in surviving quiescent cells, in a scenario when the very few surviving cycling cells were unable to sustain an increase of the overall tumor cell population.

A different model was required to fit response Type B, characterized by high cell kill with fast dying rate and by a period of time when quiescent cells were recruited into proliferation. Cell recruitment from quiescence into proliferation can be due to increased availability of nutrients or reoxygenation of hypoxic regions [34–36], in the first days of therapy. In a single case (patient L1) recruitment was observed later, in keeping with the traditional view that accelerated repopulation occurs weeks after the initiation of radiotherapy [37]. However for patient L1 an alternative model with a subpopulation of resistant cells, similar to response Type A, cannot be ruled out, despite that the fit with the recruitment model was somewhat better.

Response Types C and D, characterized by a low percentage of cells killed per fraction, were found in the remaining six cases of our series. In these cases the treatment was apparently less effective, but the correspondence of the measured volume with the tumor volume may be questioned in some of these cases. In patient L2, the presence of fibrotic tissue after neoadjuvant chemotherapy would explain why there was no further decrease in volume during RT. A similar pitfall was underlined by Bosmans et al. who explained an unexpected absence of decrease in the volume during the first 2 weeks of radiotherapy in chemotherapy-pretreated patients, despite an accelerated course [2] and contrary to two other studies [4, 7], by hypothesizing that cell death was offset by treatment-induced inflammation which increased the volume. These examples suggest that delivery of neoadjuvant chemotherapy and the resulting tumor appearance on CT may not correlate well with the actual tumor volume. Some explanations for this could be treatment-related fibrosis, atelectasis, or other treatment-related effects [38]. Incorporating other imaging modalities such as PET/CT may also be useful for improving the models.

For what concerns the other Types C and D responses, misinterpretation of the volume measurements is possible at least for patients S3 and S5. In the case of S3, fibrosis is suspect for the long-term datum, which is crucial for modeling of this case. Patient S5 had a biopsy proven recurrence after receiving 5000 cGy in 250 cGy fractions. The patient also received neoadjuvant, concurrent, and adjuvant erlotinib (150 mg) and bevacizumab (15 mg/kg) for this course of RT. The effect of chemotherapy was not included in modeling. Moreover, it is possible that fibrotic tissue from the prior course of RT and/or neoadjuvant chemotherapy could account for the lack of volume reduction during the present course of RT. Nevertheless, approximately one month later the volume had decreased by 22%.

The fact that for group S there is a visible tumor during the two-week course of treatment is both reassuring and perplexing. It is reassuring because group S having a visible lesion will benefit from more accurate targeting with image guided radiation therapy (IGRT) and gating techniques. It is perplexing because despite a biologically equivalent dose in 2 Gy fractions (EQD_2_) of 66.7 Gy_10_^2^ delivered (10 Gy × 4 at the time of the last CT) in a short two-week period, 5 of 12 patients in this group were classified as poor responders (response Types “C” and “D”).

Takeda et al. analyzed 63 patients treated with 10 Gy × 5 fractions and the 3-year local control in patients with Stages 1A and 1B being 93% and 96% [39]. Therefore, the lack of an early response in some of our group S patients may not translate to a subsequent poor local control. The apparent lack of response could be attributed to treatment-related fibrosis, which is a common clinical explanation for these CT findings. However, without a biopsy it is difficult to determine if there was residual disease.

The observation that quiescent cells responded poorly to radiotherapy suggests that a single fraction treatment may be suboptimal compared to a short hypofractionated course. The former would treat a larger proportion of “resistant” quiescent cells, while the latter would have induced cells into the very radiosensitive proliferation phase by the second fraction. In addition, higher doses than expected may be required in the single dose setting to compensate for the radioresistant quiescent cells.

This type of modeling and treatment response characterization may provide useful insights when trying to understand what is happening at the cellular level or comparing treatments. Type B, high kill and fast dying rate, tumor response would be ideal from a palliative perspective in most situations. This analysis underlines the difficulty of predicting the tumor's long-term behavior based on the first days of volume measurements because some effects (e.g., resistance) may actually emerge after a long time, being totally undetectable in the first days. Statistically, it would be possible to estimate a probability of outcomes within each response type, but this would require a higher number of patients than those included in this study.

## 5. Conclusion

The models used in this study performed well in fitting the available dataset. According to the values of parameters corresponding to cells killed and cells dying, patients were grouped in four response types. The models provided useful insights into the possible underlying mechanisms responsible for the radiotherapy tumor volume response. Further studies are necessary to better understand the clinical implications of the four response types.

## Figures and Tables

**Table 1:** Individual patient, tumor, treatment, and tumor response characteristics. Patient IDs starting with an “L” had a long RT course while those starting with an “S” had a short one.

Patient ID	Sex	Age	Pathology	Total dose (Gy)	Fx. (Gy)	BED_10_ (Gy)	Days to finish RT	Chemo
L1	M	68	SCCA	75	2.5	93.8	46	No
L2	M	78	ACA	75	2.5	93.8	43	Yes*
L3	F	75	SCCA	75	2.5	93.8	44	No
L4	F	72	ACA	80	2.5 (50 Gy) 2 (30 Gy)	98.5	70	No
L5	M	69	ACA	66.6	1.8	78.6	55	No

S1	F	65	SCCA	50	10	100.0	12	No
S2	F	66	ACA	50	10	100.0	9	No
S3	F	68	other^†^	50	10	100.0	9	No^‡^
S4	F	61	NSCLC	50	10	100.0	11	No
S5	M	70	ACA	50	10	100.0	12	Yes^§^
S6	M	85	ACA	50	10	100.0	12	No
S7	F	69	SCCA	50	10	100.0	12	No
S8	M	73	ACA	50	10	100.0	9	No
S10	F	74	NSCLC	50	10	100.0	9	No
S11	F	82	Other^#^	50	10	100.0	11	No
S12	M	74	SCCA	50	10	100.0	9	No
S13	M	66	SCCA	50	10	100.0	9	No

Abbreviations:

ID: identifier; M: male; F: female; Fx.: daily fraction dose; BED_10_: biologically effective dose, *α*/*β* ratio of 10; SCCA: squamous cell carcinoma; ACA: adenocarcinoma; NSCLC: nonsmall cell lung cancer, not otherwise specified; RT: radiotherapy; chemo: chemotherapy.

*Three cycles of neoadjuvant paclitaxel (200 mg/m^2^) and carboplatin (AUC 5).

^†^Clinical history consistent with metastatic breast adenocarcinoma.

^‡^Patient completed chemotherapy for breast cancer 5 months prior to radiotherapy.

^§^Biopsy proven recurrence after receiving 50 Gy in 2.5 Gy fractions. Received neoadjuvant, concurrent, and adjuvant erlotinib (150 mg) and bevacizumab (15 mg/kg) for this course of RT.

^#^Atypical cells suspicious for malignancy; serial CTs, PET/CT SUV ≥ 2.5, and clinical history consistent with primary lung malignancy.

**Table 2:** Parameter comparison for the four tumor growth and therapy efficacy models.

Model	Parameter										
	*Td *	*GF *	*T*_pot_	*γ*	*γ*_rec_	*K*	*Kq*	*Kp*	*D*	Rini	Rind
M: minimal	●		●			●			●		
St: standard	●	●	●	●			●	●	●		
REC: recruitment	●	●	●	●	●		●	●	●		
RES: resistant	●	●	●	●		○	○	○	●	○	○

●: Essential model parameter.

○: Model parameter options to be selected. RES model uses either parameter *K* or *Kp* and *Kq* and either Rini or Rind.

Abbreviations:

*Td*: doubling time; *GF*: growth fraction; *T*_pot_: tumor potential doubling time; *γ*: rate at which quiescent cells reenter the cycle (if *γ*rec is used, this will be the pretreatment rate); *γ*_rec_: recruitment rate *γ*; *K*: fraction of cells killed by a single fraction of RT; *Kq*: fraction of quiescent cells killed by a single fraction; *Kp*: fraction of proliferating cells killed by a single fraction; *D*: dying rate; Rini: subpopulation of resistant cells; Rind: fraction of cells that becomes resistant after each RT exposure.

**Table 3:** Individual patient, tumor, treatment, and tumor response characteristics. Patient IDs starting with an “L” had a long RT course while those starting with an “S” had a short one. Best fit values of model parameters with likelihood-based confidence ranges in square brackets.

Pt. ID	Day 0 volume (cc)	Model	Response type	*Td* (days)	*GF *	*T*_pot_ (days)	*γ*	*γ*_rec_	*K* (%)	*Kp* (%)	*Kq* (%)	*D*	Rini	Rind	Mean Δ%
L1	8.5	M Res	B	200		5.6			15 [14–16]			0.5 [0.46–0.95]	0.20 [0.19–0.21]	3.4 [3.1–3.7]	8.0
		St Rec		200	0.15	13.9	0.01	0.10* [0.08–0.11]		29 [28–30]	1 [0.5–2]	0.56 [0.47–0.71]			7.0
L2	13.2	M	C	100		9.3			1.8 [1-2]			0.2 [0.2–1]			3.5
L3	4.6	St Rec	B	400	0.05	27.8	0	0.37^‡^ [0.19–0.81]		49 [47–50]	1 [0.5–2]	0.6 [0.48–1]			10.2
L4	161.1	M Res	A	1000		5.5			10 [9–12]			0.3 [0.25–0.4]	0.31 [0.28–0.33]	3.2 [2.7-3.7]	4.0
L5	1.8	M Res	A	150		5.6			16 [14–33]			0.35 [0.2–0.7]	0.36 [0.32–0.39]	6.2 [5.8-6.8]	5.1
		St Rec		150	0.25	5.6	0	0.02^†^ [0.02–0.02]		7 [6-7]	1 [0.5–2]	0.36 [0.25–0.42]			5.4

S1	3.2	St	A	25	0.06	22.8	0			90 [90–100]	5 [5–9]	0.2 [0.2–0.28]			8.4
S2	12.1	St Rec	B	25	0.10	14.3	0	0.26^§^ [0.05–0.54]		90 [86–99.9]	1 [0–6]	1 [0.85–0.9999]			6.8
S3	2.0	M	D	250		5.6			8 [5–11]			0.999 [0.25–1]			6.6
S4	2.0	M	C	ND		ND			25 [5–44]			ND			4.0
S5	23.5	M	C	150		8.4			9 [5–12]			0.2 [0.2–0.4]			3.7
		St		150		8.4				90 [38–100]	2 [0.01–6]	0.2 [0.1–0.62]			5.1
S6	8.1	M	D	45		5.6			13 [11–21]			1 [0.57–1]			6.6
S7	4.7	St	A	30	0.06	23.6	0			99 [98–100]	23 [23–25]	0.2 [0.16–0.29]			9.2
S8	1.2	St Rec	B	1000	0.05	27.7	0	0.48^§^ [0.27– 0.68]		65 [59–85]	1 [0–7]	0.9 [0.44–1]			7.2
S10	3.2	St	A	15		9.7	0.01			99 [85–100]	23 [20–25]	0.2 [0.15–0.31]			13.1
S11	1.7	M	D	15		5.8			22 [20–23]			0.99 [0.95–1]			23.9
S12	0.7	St Rec	B	75	0.05	28.0	0	0.74^§^ [0.23–0.79]		76 [74–95]	0.01 [0.01–4]	0.75 [0.1–0.97]			7.2
S13	4.1	St	A	1000		5.5				90 [57–99]	53 [53–99]	0.2 [0.2–0.25]			14.6

Abbreviations:

Pt.: patient; Mean Δ%: average percent difference between data and fit; minimal (M) and standard (St) models are defined in the text, with the respective growth parameters (*Td*, *GF*, *T*_pot_, and *γ*) and killing parameters (*K*,*Kp*, *Kq*, and *D*). For the resistance models, two alternative resistance models (Res) were considered: (i) initial resistance, with parameter Rini (fraction of resistant cells at *t* = 0); (ii) induced resistance, with parameter Rind (probability of induced resistance per fraction delivered). The recruitment model (Rec) is characterized by the parameter *γ*rec (fraction of quiescent cells recruited into proliferation per day).

Response types: A: “high” kill and “slow” dying rate; B: “high” kill and “fast” dying rate; C: “low” kill and “slow” dying rate; D: “low” kill and “fast” dying rate.

*Recruitment from 4th week after treatment.

^†^Recruitment from 3rd week after treatment.

^‡^Recruitment on days 1–13 after treatment.

^§^Recruitment on days 1–3 after treatment.
